# Cultural efficacy predicts body satisfaction for Māori

**DOI:** 10.1371/journal.pone.0253426

**Published:** 2021-06-23

**Authors:** Carla Houkamau, Samantha Stronge, Isaac Warbrick, Kiri Dell, Jason Mika, Jamie Newth, Chris Sibley, Khanh Linh Kha

**Affiliations:** 1 Department of Management and International Business, University of Auckland, Auckland, New Zealand; 2 Department of Psychology, University of Auckland, Auckland, New Zealand; 3 Taupua Waiora Research Centre, Faculty of Health & Environmental Sciences, Auckland University of Technology, Auckland, New Zealand; 4 School of Management, Massey University Manawatū, Palmerston North, New Zealand; College of Medicine and Sagore Dutta Hospital, INDIA

## Abstract

This paper examines the relationship between body mass index (BMI), self-esteem and self-reported confidence and capability in expressing oneself culturally as Māori (cultural efficacy) for 5,470 Māori who participated in Te Rangahau o Te Tuakiri Māori me Ngā Waiaro ā-Pūtea | The Māori Identity and Financial Attitudes Study (MIFAS) in 2017. Adjusting for demographics, self-reported health, education and socio-economic status, we found that a higher BMI was associated with lower body satisfaction and self-esteem. However, higher scores on cultural efficacy were associated with higher levels of body satisfaction and self-esteem for respondents. Furthermore, the negative association between BMI and both body satisfaction and self-esteem was weaker for those with higher cultural efficacy. This held for BMI scores of 25, 30, and 35+. While our data suggest higher cultural efficacy may directly or interactively shield Māori from developing lowered self-esteem typically associated with higher BMI in Western populations, further research, using more comprehensive measures of body satisfaction should explore the extent to which Māori may find the Western “thin ideal” personally desirable for their own bodies.

## Introduction

Body image may be defined as one’s perceptions of, and attitudes towards, one’s body, especially physical appearance [1]. Body dissatisfaction stems from inconsistency between one’s perceived and desired body size and shape [2], with norms for body size often based crudely on body mass index (BMI). Although thinness has not always been idealised [3], historically, women have experienced more social pressure to be thin, but men are not immune [4]. Cultural factors significantly influence idealised body weight, size and shape [5–8]. Much of the academic literature on body dissatisfaction emerges from the United States [9] and the United Kingdom [10], and the cultural reference point is Western and ethnically European [6, 11]. Internationally, it is known that some cultures accept or even desire larger body sizes [12, 13].

Western attitudes are an approximate but insistent commonality across developed countries primarily, affecting many ethnicities, particularly via the media. Importantly, people from non-European cultures may identify strongly and positively with their non-Western culture, identity and traditional body ideals. Modern Western socio-cultural beliefs and practices both privilege thinness and stigmatise obesity [14–17]. New Zealand’s Western-dominated culture [18] generally esteems slenderness [19]. The country is not culturally homogenous, however; and at about 17% of the population, Māori (the indigenous people of New Zealand) are the second-largest ethnic group after Pākehā (New Zealanders of European descent) at 72% [20]. Pākehā primarily descend from the settlers from the United Kingdom who colonised the country formally from 1840 and soon dominated politically and economically. Despite two centuries of co-existence, many Māori maintain distinctive cultural values [21].

New Zealand has one of the highest rates of people who are overweight and obese among OECD nations, with one in three adults being classified as obese [22]. Ethnic patterns of weight are marked: 66.5% of Pacific peoples, 48.2% of Māori, 29.1% of European/Other and 13.8% of Asian adults were reported as obese by the Ministry of Health [23] using data from the New Zealand Health Survey 2018/19 [24]. Notably too, Māori experience worse mental health than non-Māori and, excepting Pacific peoples, have higher official rates of overweight and obesity [25]. Māori are also more likely than Pākehā to be exposed to forms of discrimination, such as racism, and are more likely to be portrayed negatively in the media [26, 27]. In light of this context, the psychosocial correlates of body satisfaction and self-esteem are important considerations for Māori, given that poor self-concept and body dissatisfaction have a negative impact on psychological well-being.

Some commentators have proposed the Western (mainstream/Pākehā) cultural ideal of thinness is not shared or internalised–that is, personally adopted and accepted–by all Māori [28], and empirical research variously suggests Māori can be impervious or susceptible to it. By applying a measure of cultural efficacy with a large sample from a uniquely comprehensive indigenous dataset, this study expands understandings of Māori body image and body satisfaction in relation to the varying degrees of strength and confidence in expressing identity as Māori. We also explore the extent to which weight links to higher self-esteem for Māori, and therefore how cultural connection and cultural efficacy may promote this population’s overall mental well-being. The mental health toll of judging oneself against the thin ideal of beauty is our main concern. However, on the flip side of that psychologically damaging aesthetic, we appreciate the genuine health risks associated with lifestyle factors often linked to being overweight–sedentariness, diet and so forth.

The next sections review the literature on Western ideals of thinness and the associated psychological implications, possible protective effects of non-Western culture, and the situation of Māori. After setting out the guiding hypotheses, we present our method, followed by the results and discussion sections. The discussion includes consideration of how cultural efficacy and other cultural insights can be sensitively leveraged in reverse to reduce those lifestyle factors associated with diminished health (and usually excess weight) among Māori.

### Self-esteem, body satisfaction and mental health

Self-esteem is one’s positive or negative attitude towards oneself and one’s evaluation of one’s own thoughts and feelings overall concerning oneself [29]. Self-esteem closely relates to body satisfaction [30–33]. Higher BMI correlates with lower self-esteem in Western populations [34, 35]. Effects run in both directions [36], with body satisfaction lifting self-esteem and self-esteem fostering body acceptance [30]. Wishing for an unattainably slimmer body can beget frustration, despondency, and unhealthy behaviours like maladaptive exercise and eating (e.g., [37–39]), including binging and compensatory eating [40]. While women suffer the psychological toll more, men are not immune. For example, Warbrick et al. [41] observed that the motivation to continue exercise among a cohort of Māori men was negatively impacted if significant weight loss was not achieved after 12 weeks. Men internalising social pressures may desire not only slim, but also athletic, muscular physiques low in body fat [42, 43]. Western standards of beauty are unachievable for many, and research consistently links internalising the thin ideal with depressive symptoms and other psychological disturbances among diverse populations [44]. Thus, extreme body dissatisfaction becomes a mental health issue. As such, we aim to investigate the associations between weight and both body satisfaction and self-esteem to examine both the specific and broader effects on mental well-being associated with not meeting Western body mass standards.

### Protective factors: Non-Western culture and ethnicity

On the one hand, the quest for extreme thinness, and its toll, are spreading to non-Western cultures [45]. Take three examples: First, growing research suggests that Asian women desire body types promoted by Western beauty standards [46, 47], bringing higher body dissatisfaction [48]. Second, while traditional Latino culture idealises a larger physique [49, 50], several studies report body dissatisfaction among Latina women at levels almost equal to White American women, evidencing an internalised Western beauty standard (see [51] for a review). Third, although Polynesian cultures traditionally value larger body sizes as healthy and attractive, Craig et al. [52] found evidence for the impact of the Western thin ideal among Rarotongans (Cook Islanders). Eighty-three females and 49 males in a Rarotongan village were matched with a sample of Australians for sex, age and BMI. The Cook Island females showed similar attitudes towards body size as Australian women, both groups specifically desiring a slimmer physique and smaller body size.

On the other hand, the thin Western ideal is accepted neither uncritically nor universally [5, 7, 53]. For example, in a survey of over 4,000 indigenous and Anglo-European adolescents in Australia, indigenous (Aboriginal/Torres Strait Islander) males and females 12–16 years old (*n* = 333) were significantly less likely to desire weight loss and more likely to desire weight gain than White Australians (*n* = 4367) [54]. Extending this, but homing in on socio-cultural and religious factors, Muslim adolescent girls attending madrassa (Islamic religious school) and wearing hijab reported higher body satisfaction than non-hijab-wearing Muslim adolescent girls who followed contemporary, Western-influenced fashion trends and had lower BMIs [45]. In other studies, compared with White American women, African-American women consistently viewed larger body sizes more favourably [55], and a meta-analysis of 98 U.S. studies published over 1960–2004 found White American women reported greater body dissatisfaction than African-American (although the difference was small) [11].

This second set of three examples suggests that culture may act as a protective factor. But how exactly? Rakhkovskaya and Warren [56] observe specifically that strong ethnic identity may protect against eating disturbances by helping ethnic minority women reject mainstream Western media values and the thin ideal because they feel connected to and secure in their own ethnic identity. Relative acceptance of Western standards of beauty may depend on how far individuals have internalised Western standards [57, 58]. On the other hand, cultural pride, values, and beliefs that promote a different standard offer an alternative to dominant discourses [59]. Thus, in another of the many U.S. studies, African-American women focus group participants rejected the idealisation of thinness for a multifaceted beauty ideal of personal style, self-care, and spirituality [60]. This may speak to aspects of their culture basing attractiveness on unique qualities, “inner beauty”, self-confidence and style [61, 62]. Similarly, and contrary to some of the studies above (reviewed by Stokes et al. [51]), a decade earlier Warren et al. [63] had found Spanish women (living in Spain) and Mexican-American (defined as persons of Mexican descent living in the United States) women to be as aware of the thin ideal as European Americans *(sic)*, but less likely to report internalising it. Overall they found that American women had overall higher body dissatisfaction than the two other groups (which did not differ significantly) and observed that the Mexican and Spanish culture’s female ideal of a larger, curvy physique might facilitate accepting larger bodies.

Turning to New Zealand, some evidence supports claims [28, 64] that the thin Western ideal does not appeal to Māori. It is possible that Māori have a different reference point for being overweight. For example, Metcalf et al. [65] found that Māori and Pacific Islanders classified as “moderately overweight” and “very overweight” by BMI standards regarded their weight as healthy. Alternatively, a strong connection to Māori culture, or understanding of how to express the self culturally as Māori, might buffer against Western body ideals, even if Māori share Pākehā reference points through mainstream New Zealand media. Finding Māori less concerned about body mass than Pākehā, Talwar et al. [66] concluded that more significant affiliation with Māori ethnic identity significantly lowers weight concerns, which is considered an essential element of body esteem. However, some research finds similar body image perceptions among Māori and Pākehā [67]. For example, among university students, Turangi-Joseph [68] uncovered no significant differences in body image or body dissatisfaction and dieting behaviours or disordered eating attitudes. Similarly, Ngamanu [69] detected no differences in body dissatisfaction and eating pathology, regardless of ethnic attachment. This absence of a clear, consistent link between cultural identity and body dissatisfaction may reflect differing conceptualisations and measures of cultural identity used in different studies, and therefore clarity around which aspects of cultural identity are relevant for body image satisfaction is required.

### Cultural efficacy

In the context of colonisation, indigenous peoples internationally have sought to counteract histories of cultural assimilation and marginalisation by emphasising learning about their own traditional cultural practices, including language, to affirm a positive ethnic identity [70, 71]. The reasoning is that understanding their own culture will instil in members the pride and self-worth to cope with and psychologically buffer discrimination and prejudice from wider society [72]. Research supports this rationale [73, 74], and on that basis Māori writers have argued that Māori need to learn about their language, heritage and culture to fortify their personal self-esteem and promote mental and physical health [75–79]. Following this, our rationale was to explore the extent to which cultural efficacy provides a protective role for Māori, mitigating the impact of higher BMI on self-esteem.

The tool used to measure cultural efficacy is the Cultural Efficacy and Active Identity Engagement Scale, which is a component of the Multidimensional Model of Māori Identity and Cultural Engagement (MMM-ICE). Created in 2010, the MMM-ICE is a public-domain self-report scale currently in its third iteration–the MMM-ICE3. Several papers describe the inception, development and validation of the MMM-ICE scale (see [80–83]). The first iteration, originated by Houkamau and Sibley (see [80]), utilised exploratory factor analysis from an online sample of Māori (*n* = 270) to identify six interrelated aspects or “subscales” of Māori identity. The original six dimensions were Group Membership Evaluation; Interdependent Self-Concept; Cultural Efficacy and Active Identity Engagement; Spirituality; Socio-Political Consciousness; and Authenticity Beliefs [80, 84]. Houkamau and Sibley [81] then updated the survey with MMM-ICE2 to include a seventh subscale, Perceived Appearance, reflecting the worse discrimination reported by Māori identifying as solely of Māori ethnicity, who “look” more Māori (see [82], p. 479). The MMM-ICE survey has been further refined to more accurately capture the factors hypothesised as being part of Māori identity. Changes in the MMM-ICE3 include an eighth subscale: Whānau Efficacy (Family Efficacy). This comes from the Māori concept of whānau or extended family and is utilised to explore the role of whānau/family connectedness for well-being among Māori. The MMM-ICE3 also reduced and slightly reworded certain items across all factors for brevity and clarity and to minimise respondent burden. Each scale now comprises four or five items, which respondents rate from 1 (strongly disagree) to 7 (strongly agree). A full account of developing the new subscale, including the operational definitions of the full eight MMM-ICE3 dimensions/subscales, has recently been published (see [85, 86]).

In this study we measure cultural efficacy using the MMM-ICE Cultural Efficacy and Active Identity Engagement Scale, which measures how confident individuals feel when they are among other Māori or in situations which require the active expression of Māori customary knowledge (see Questionnaire Measures for specific items).

### Summary and guiding hypotheses

While data from various ethnic groups suggest that ethnic identity may prevent individuals from internalising Western body image ideals, ethnic identity is multi-dimensional and it is not clear precisely which aspects of ethnic identity may predict body image satisfaction or wellbeing for Māori. We therefore ask: Does cultural efficacy buffer Māori men and women from the Pākehā-associated thin ideal? Although evidence indicates that some Māori women desire smaller bodies, a strong connection to Māori culture could buffer this, acting either directly or interactively as a moderator between BMI and body satisfaction, and BMI and self-esteem. Consequently, we hypothesise that a confidence in expressing the self culturally as Māori may help some Māori reject extreme thin beauty ideals.

## Method

The research was approved by the University of Auckland Human Participants Ethics Committee for the period 16 May 2016 until 16 May 2022. Reference Number: 017154.

### Sampling and procedure

This paper draws on the nationwide Te Rangahau o Te Tuakiri Māori me Ngā Waiaro ā-Pūtea | Māori Identity and Financial Attitudes Study (MIFAS). MIFAS is a pen-and-paper questionnaire posted to a random sample of 100,000 people on the New Zealand electoral roll (whether “general” or “Māori” roll–the voter’s own choice) claiming Māori descent. Hence all respondents already self-identified as Māori. Data come from the first wave of respondents in 2017–2018. MIFAS comprises over 340 individual items, takes approximately 30–45 minutes to complete and embeds a short, 40-item version of the MMM-ICE3 [85, 87]. Information about the MIFAS sample, methods, response rate (approximately 7%) and representativeness has been provided elsewhere [85].

### Questionnaire measures

#### Cultural efficacy

Although all participants already identified as Māori, we wanted to quantify the strength and confidence expressing the self culturally as Maori. Participants completed the Cultural Efficacy and Active Identity Engagement (CEAIE) Subscale (α = .80) from the MMM-ICE3 [85]. The subscale measures the extent individuals perceive they have the personal resources to engage appropriately with other Māori in Māori social and cultural contexts. Respondents indicated (dis)agreement with five items on a Likert scale, from 1 (strongly disagree) to 7 (strongly agree). Statements were: 1) “I do not know how to behave on a marae [traditional meeting place]”; 2) “I try to kōrero (speak) Māori whenever I can”; 3) “I know how to behave the right way when I am on a marae”. 4) “I can’t do Māori culture or speak Māori”, and 5) “I have a clear sense of my Māori heritage and what it means for me.”

#### Self-esteem

To measure self-esteem, we used the following three items from the Rosenberg Self-Esteem Scale [29]: 1) “On the whole, I am satisfied with myself”, 2) “On the whole, I take a positive attitude toward myself”, and 3) “On the whole, I am inclined to feel that I am a failure” (reverse coded). Participants circled a number from 1 (very inaccurate) to 7 (very accurate). Averaging the items gave a scale score (α = .69).

#### Body satisfaction

Body satisfaction was measured using participants’ responses to a single item that was devised for the New Zealand Attitudes and Values Study [88]: “I am satisfied with the appearance, size and shape of my body” [89, 90]. Responses used a Likert scale rating from 1 (very inaccurate) to 7 (very accurate).

#### Body mass index

BMI was calculated using self-reported height and weight data. The formula is BMI = kg/m^**2**^ where kg is weight in kilograms and m^**2**^ is height in metres squared. A BMI in the range 18.5–24.9 is considered normal or healthy weight, 25–29.9 is considered overweight, and 30 and above is considered obese. Overweight and obesity increase the risk of certain chronic diseases [91–98].

#### Covariates

We controlled for eight demographic variables: gender, age, sole Māori ethnicity, education, disability status, deprivation, socio-economic status, and household income. For gender, we asked, “What is your gender?” and respondents wrote any answer of their choosing (coded as 0 for women, 1 for men). For age, we asked for the participant’s date of birth and calculated age in years. For sole Māori ethnicity, we asked, “Which ethnic group(s) do you belong to?” and respondents could select one or more from “New Zealand European, Māori, Samoan, Cook Island Māori, Tongan, Niuean, Chinese, Indian, Other (such as Dutch, Japanese, Tokelauan)” and could specify another ethnicity. We coded whether or not participants identified solely as Māori, or as Māori and one or more other ethnicities (0 multiple ethnicities, 1 solely Māori). For education, we asked, “What is your highest level of education?” (open-ended). Responses were then coded according to the New Zealand Qualifications Framework (NZQF) codes. The NZQF is divided into 10 levels, and covers a range of qualifications from certificates to doctoral degrees. The levels are based on how complex the learning is, with a level 1 certificate the least complex (see [99]). We coded our data on a scale from 0 (no qualification) to 10 (doctoral degree). For disability status, we asked, “Do you have a health condition or disability that limits you and that has lasted for 6+ months?” (0 no, 1 yes).

Deprivation was measured using the New Zealand Index of Deprivation, which uses census information to assign a decile-rank index from 1 (least deprived) to 10 (most deprived) to each neighbourhood “meshblock” unit. Deprivation represents a wide range of factors such as income, home ownership, housing quality, family structure, and access to facilities such as transport and the internet [100]. Socio-economic status was measured using the New Zealand Socio-Economic Index, which uses age, education and occupation to generate a socio-economic status score on a scale from 10 (low) to 90 (high) [101]. Household income was also assessed by asking, “Please estimate your total household income (before tax) for the year” (open-ended). Similar to papers examining economics and well-being (e.g., [102]), we use logarithmic household income in the analyses. This accounts for the fact that the difference between an annual income of $10,000 or $20,000 is much larger than the difference between an annual income of $100,000 or $110,000.

#### Participants

Of the 7,019 participants who completed the MIFAS, 5,470 provided complete responses to the measures of cultural efficacy and BMI and were included in the analyses. The mean age of the participants was 48.06 years (*SD* = 14.64; range = 18–83), and 60.3% of the sample identified as female (*n* = 3,296). In terms of BMI, 23.8% of the sample were classified as normal or healthy weight, 31.9% as overweight, and 44.3% as obese. Descriptive statistics and bivariate correlations for all variables are presented in Table 1.

**Table 1:** Bivariate correlations and descriptive statistics for all variables.

	1	2	3	4	5	6	7	8	9	10	11	12
1. Body satisfaction	-											
2. Self-esteem	.471	-										
3. Gender (0 female, 1 male)	.152	.053	-									
4. Age	.134	.198	.123	-								
5. Sole Māori ethnicity (0 no, 1 yes)	.064	.080	.037	.210	-							
6. Deprivation (1 low, 10 high)	.002	-.023	-.025	.042	.264	-						
7. Education (0 low, 10 high)	-.006	.054	-.101	-.154	-.151	-.161	-					
8. Disability (0 no, 1 yes)	-.049	-.095	.038	.213	.075	.109	-.100	-				
9. Socio-economic status (10 low, 90 high)	-.022	.098	-.143	-.002	-.114	-.173	.589	-.074	-			
10. Household income (log)	-.020	.087	.019	-.050	-.118	-.215	.212	-.152	.221	-		
11. BMI	-.317	-.086	.029	.085	.169	.191	-.089	.115	-.049	-.062	-	
12. Cultural efficacy	.077	.168	-.104	.001	.334	.246	.097	.020	.064	-.041	.143	-
Mean	4.31	5.47	.40	48.06	.37	6.31	4.02	.25	48.60	10.94	30.27	4.79
*SD*	1.86	1.20	.49	14.64	.48	2.85	2.75	.44	16.99	1.49	7.27	1.41

Note: Correlations above.26 are significant at *p* < .05; correlations above.30 are significant at *p* < .001.

#### Analytic plan

To test the interaction between cultural efficacy and BMI, we mean-centred both variables and constructed an interaction term by multiplying the two mean-centred variables together. We conducted multiple regressions predicting body satisfaction and self-esteem by mean-centred BMI, mean-centred cultural efficacy, the interaction term, and the covariates. We additionally conducted these analyses with covariates removed, which did not significantly change the results. These results are presented in the S1 File. Missing data in the covariates (gender, age, sole Māori ethnicity, education, disability status, deprivation, socio-economic status, and household income) were estimated using Rubin’s [103] procedure for multiple imputation, with parameter estimates averaged over 100 datasets (thinned using every 100th iteration).

## Results

**Fig 1:**
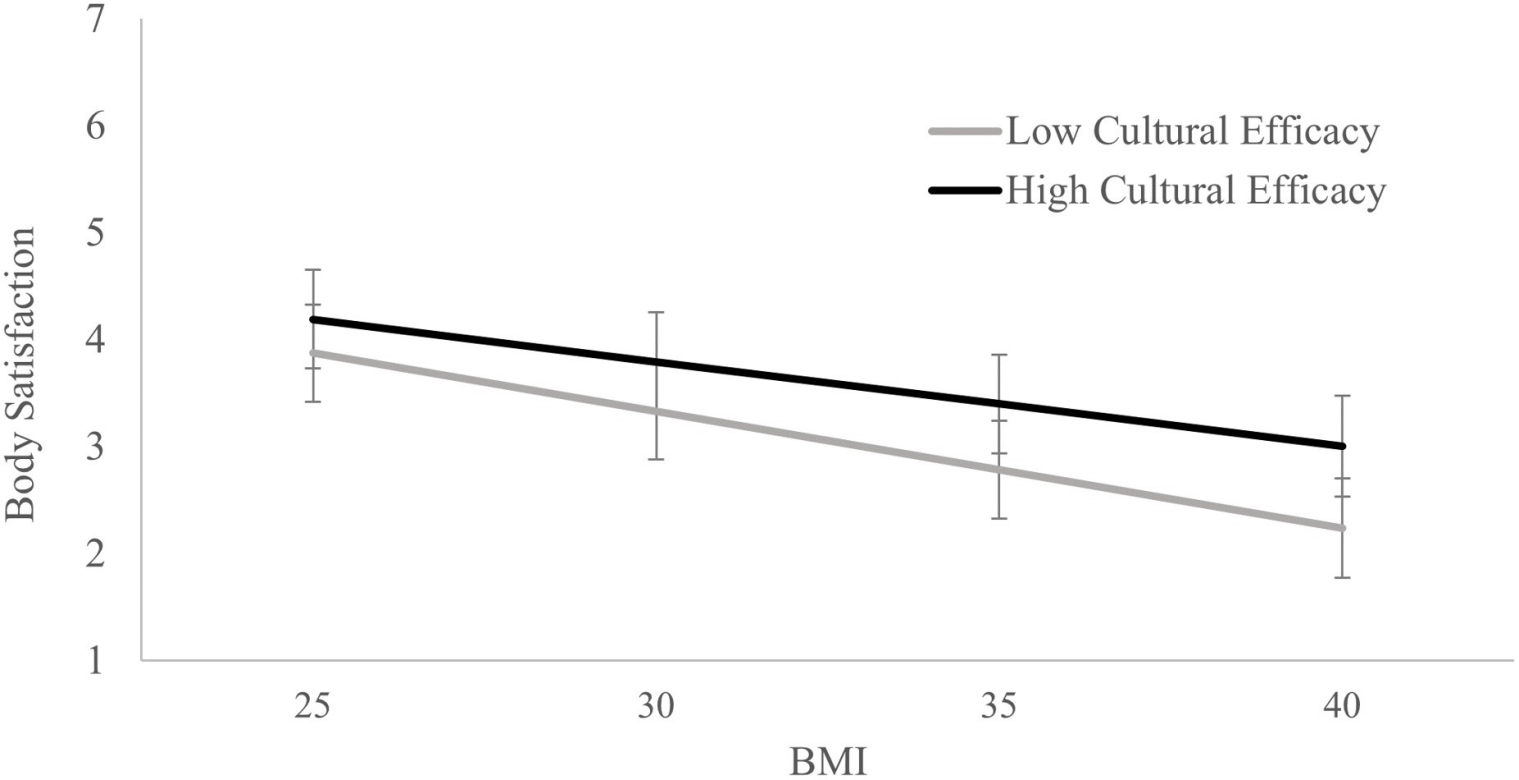
Estimated levels of body satisfaction for high (+1 SD) and low (-1 SD) levels of cultural efficacy and identity engagement and different BMIs. Error bars represent the 95% confidence intervals.

**Fig 2:**
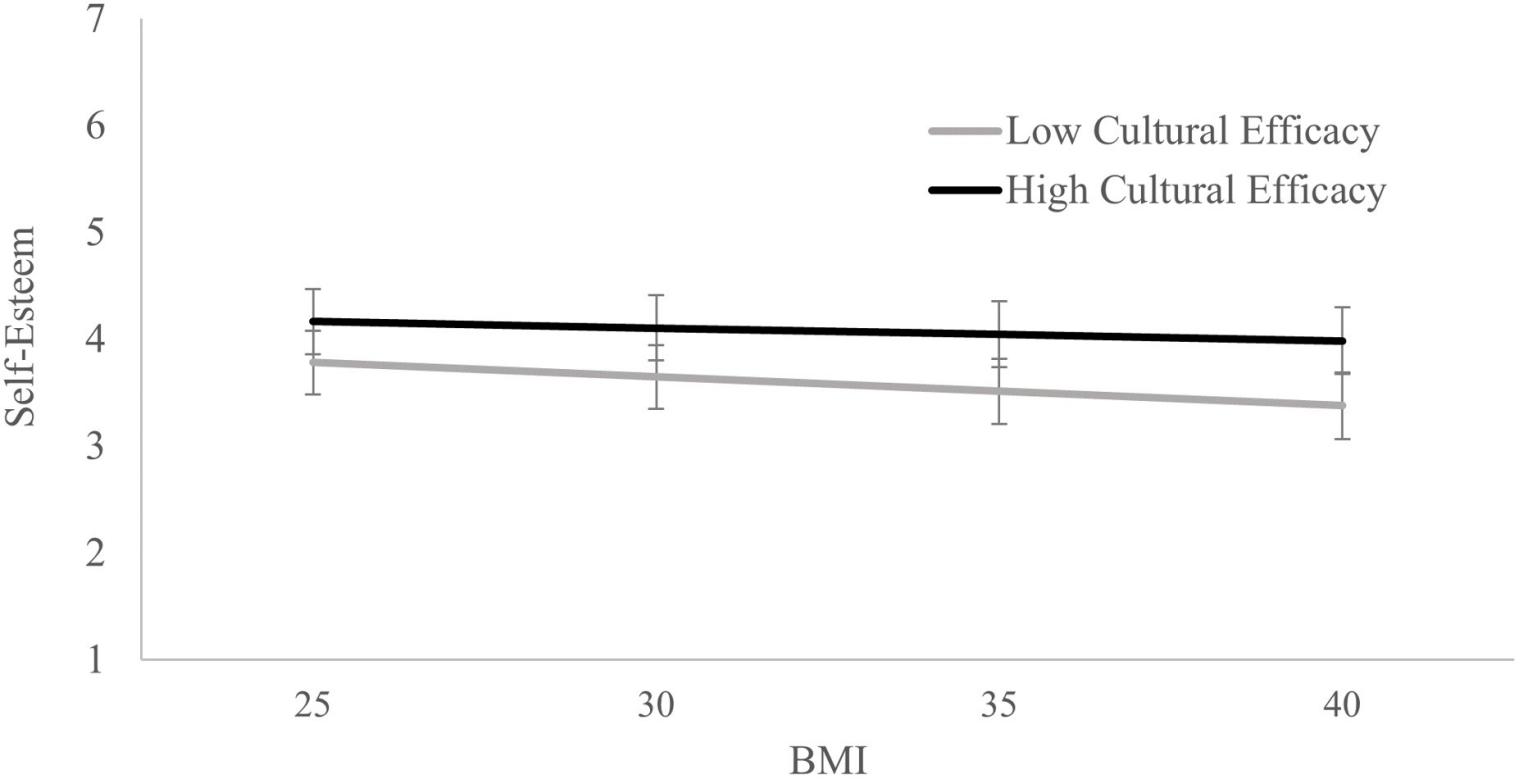
Estimated levels of self-esteem for high (+1 SD) and low (-1 SD) levels of cultural efficacy and identity engagement and different BMIs. Error bars represent the 95% confidence intervals.

The regression results are presented in Table 2. Results are also presented graphically in Figs 1 and 2. Looking first at the regression predicting body satisfaction, the results showed higher BMI was associated with lower body satisfaction, while cultural efficacy was associated with higher body satisfaction. Being male, older, identifying solely as Māori, and living in areas with higher deprivation were associated with higher levels of body satisfaction, while having a disability and higher household income were associated with lower body satisfaction. Socio-economic status and education were not significantly associated with body satisfaction.

**Table 2:** Multiple regression predicting body satisfaction and self-esteem by BMI, cultural efficacy, and covariates.

	Body Satisfaction							Self-Esteem						
	*b*	*β*	*SE*	*t*	*p*	95% CI		*b*	*β*	*SE*	*t*	*p*	95% CI	
BMI	-.094**	-.367	.003	-28.511	< .001	-.100	-.088	-.019**	-.118	.002	-8.835	< .001	-.024	-.015
Cultural efficacy	.168**	.127	.018	9.300	< .001	.133	.203	.165**	.192	.012	13.633	< .001	.141	.188
BMI × cultural efficacy	.011**	.057	.002	4.614	< .001	.006	.015	.005*	.044	.002	3.426	.001	.002	.008
Gender (0 female, 1 male)	.602**	.158	.048	12.508	< .001	.507	.696	.148**	.060	.032	4.618	< .001	.085	.212
Age	.019**	.149	.002	11.311	< .001	.016	.022	.019**	.227	.001	16.712	< .001	.017	.021
Sole Māori ethnicity (0 no, 1 yes)	.143*	.037	.053	2.672	.008	.038	.247	.035	.014	.036	0.974	.330	-.035	.105
Deprivation (1 low, 10 high)	.018*	.027	.009	1.969	.049	.000	.036	-.009	-.022	.006	-1.520	.129	-.021	.003
Education (0 low, 10 high)	.006	.009	.011	0.573	.567	-.016	.028	.001	.003	.007	0.158	.874	-.013	.016
Disability (0 no, 1 yes)	-.246**	-.058	.055	-4.487	< .001	-.354	-.139	-.332**	-.120	.037	-9.054	< .001	-.404	-.260
Household income (log)	-.037*	-.030	.018	-2.068	.039	-.072	-.002	.050**	.062	.012	4.199	< .001	.027	.073
Socio-economic status (10 low, 90 high)	-.002	-.022	.002	-1.297	.195	-.006	.001	.004**	.061	.001	3.697	< .001	.002	.007

* *p* < .05.

** *p* < .001.

Critically, the interaction between BMI and cultural efficacy for body satisfaction was significant. The simple slopes showed that BMI had a weaker association with body satisfaction for those with high (+1 SD) cultural efficacy scores (*b* = -.079, *SE* = 004, *t* = -18.384, *p* < .001, 95% CI [-.087, -.071]) compared to those with low (-1 SD) cultural efficacy scores (*b* = -.109, *SE* = .005, *t* = -22.129, *p* < .001, 95% CI [-.119, -.099]).

For the self-esteem regression, the results showed that higher BMI was associated with lower self-esteem, and higher cultural efficacy was associated with higher self-esteem. Being male, older, having a higher household income and higher socio-economic status were associated with higher levels of self-esteem, while having a disability was associated with lower self-esteem. Identifying solely as Māori, living in areas with higher deprivation, and education were not significantly associated with self-esteem.

Furthermore, the interaction between BMI and cultural efficacy was again significant. The simple slopes showed that BMI had a weaker association with self-esteem for those with high cultural efficacy scores (*b* = -.012, *SE* = .003, *t* = -4.192, *p* < .001, 95% CI [-.018, -.006]) compared to those with low cultural efficacy scores (*b* = -.027, *SE* = .003, *t* = -8.173, *p* < .001, 95% CI [-.033, -.020]).

The interaction results are presented in Fig 1 for body satisfaction and Fig 2 for self-esteem, with levels of body satisfaction and self-esteem estimated for those with high (+1 SD above the mean) and low (-1 SD below the mean) cultural efficacy scores at BMIs of 25 and 30, 35 and 40. As noted above, BMIs 25–29.9 is considered overweight, and 30 and above is considered obese.

The figures demonstrate that those with higher than average levels of cultural efficacy tend to have higher levels of body satisfaction at all BMIs compared to those with lower than average levels of cultural efficacy. Furthermore, although a higher BMI is associated with lower body satisfaction and self-esteem, the association is weaker for those with high cultural efficacy compared to those with low cultural efficacy.

## Discussion

Our data show that Māori who scored higher on cultural efficacy recorded greater body satisfaction and greater self-esteem, even at higher BMIs, whereas those who scored lower on cultural efficacy were less satisfied with their bodies and their selves. BMI was associated with lower body satisfaction and self-esteem, however this relationship was weakened for those who reported greater cultural efficacy. As such, our data suggests greater cultural efficacy promotes psychological well-being and acceptance of body size and shape among Māori. Indeed, the further participants were from the thin ideal, the greater the role cultural efficacy played in maintaining acceptance of body size and acceptance of the self.

Māori acceptance of higher BMIs may reflect the actuality that BMI is not a definitive measure of health for Māori. Despite being the standard measure of excess fat [104], BMI does not differentiate fat from lean tissue [105], nor does it account for body frames [106]. Swinburn et al. [107] note higher levels of fat-free mass and less body fat among Polynesians (here including Māori) compared to Europeans with the same BMI. Thus, BMI may overestimate body fat levels and disease risk for Māori [108]. It seems that while weight and BMI may help identify metabolic health trends at a population level, when used at the individual level, these measures do not factor in individual differences in body composition, nor holistic Māori definitions of “health”, beyond an association with body shape and size [28].

### Limitations

The data for these analyses were taken from the MIFAS, which is a large-scale longitudinal survey that addresses a wide range of topics related to Māori identity, financial attitudes and well-being (see [85]). Māori have been found to participate in surveys at much lower rates than other New Zealanders [25, 109] and are more likely to remove themselves from survey-based studies over time [110]. Being acutely aware of this reality, every item in the MIFAS was considered very carefully to reduce response fatigue in the hope that participants would actually complete the entire survey. The sheer length and breadth of the questionnaire meant we allocated single items to measure certain variables, including body satisfaction. We appreciate this renders our analyses vulnerable to criticism from proponents of more comprehensive body satisfaction scales. Hoeppner et al. [111] propose that, in principle, a short scale might represent an improvement on the full scale, particularly in situations where there are ethical reasons to reduce scale items–such as the need to minimise participant burden in cases where surveys are very time-consuming for respondents to complete. Others acknowledge that the use of single-item measures provides a balance between practical needs and psychometric concerns when longer scales are not feasible [112–116]. Even so, we acknowledge that our single item for body satisfaction may be vulnerable to unknown biases in meaning and interpretation [117, 118]. Our study was thus necessarily limited in focusing on body satisfaction as a broadly defined construct and did not measure more specific sub-facets of body image or satisfaction. To address this limitation we recommend replicating our study with the implementation of more comprehensive measures of body satisfaction, including appearance, size and shape as separate dimensions as well as items which examine the extent to which Māori may find the Western “thin ideal” personally desirable for their own bodies.

Future research may also benefit from exploring perceptions of body satisfaction and dissatisfaction for Māori to identify cognitive and behavioural elements, as well as culturally relevant appearance ideals (see [117]).

To some extent, we were also limited by our self-esteem measure. We used three items from the original Rosenberg Self-Esteem Scale [29], which initially comprised a 10-item scale. Abbreviated versions of the Rosenberg Self-Esteem Scale have been used for several decades [119] and shown acceptable validity [120]. The three-item self-esteem scale adopted for this analysis has been used elsewhere with Māori and found to satisfactorily predict cultural efficacy (i.e., as noted, confidence in expressing the self culturally as Māori, including speaking the Māori language) [121]. Therefore, our finding is consistent with previous research suggesting both reliability and validity. Further exploration of the link between cultural connection and acceptance of one’s body and one’s self is required to substantiate the pattern we see in these data and to understand how cultural efficacy and connection interact to influence body satisfaction and self-esteem.

Self-reported data, including height and weight, were used to calculate BMI. Previous studies indicate that the correlations between self-reported and actual measured values for BMI are reasonably accurate for obesity in large cohort studies [122, 123]. However, at the same time, the accuracy of BMI based on self-reported height and weight has been criticised, as research suggests that adults, particularly older adults and women, tend to under-report their own weight and that the gap between self-reported weight and actual weight increases with obesity [124–127]. Self-reported measures of weight and height for BMI calculation were a necessity in this study, being the only option given the research method (a postal survey) and the constraints of time and resources. We note that the reliance on self-reported data is a limitation of this study, and further investigation is needed to better assess self-reported vs measured height and weight discrepancies for Māori. Importantly though, it was clear that Māori reported more body satisfaction even at higher BMIs, suggesting that even if they were judging their bodies inaccurately, the patterns we found in the data would still be the same.

The study is based on a cross-sectional design, and thus it is not possible to establish cause and effect between cultural identity and body satisfaction. Although we have controlled, as much as possible, for potential covariates, including health status, gender and age, other factors such as temperament, media consumption, and recent experiences also play an essential role in body satisfaction. In this study we have taken into account some such factors, but others were not accounted for, and this is clearly a limitation. Finally, our sample is skewed towards those who are older (average age of 48 years) than the general Māori population, where the average age is approximately 25 years according to the 2018 Census [128]. We also slightly oversample women (60% of the sample) as compared to men. We statistically adjust for both age and gender in our analyses, but note that our sample is not entirely representative of the Māori population. However, large samples of indigenous people are rare in psychological science and represent a real strength of this research.

The psychosocial correlates of obesity are essential considerations for indigenous peoples, given that poor self-concept and body size dissatisfaction negatively impact mental and emotional health. Our study is the first to use a large-scale national panel sample to examine the body satisfaction and self-esteem of Māori and how this may relate to cultural identity. The uniqueness of this sample can be considered a novel contribution to knowledge about the relationship between self-esteem and body satisfaction. For over a decade, psychology has been criticised for an (often Americo-centric) concentration on “Western, educated, industrialized, rich, and democratic (WEIRD)” populations [129, 130] and overlooking ethnic diversity in samples studied to advance disciplinary knowledge [130, 131]. The psychology corpus especially notes as a limitation the absence of work on indigenous identities and experiences worldwide [132, 133]. Thus, this study offers insight into the experience of an indigenous group rarely discussed in literature outside the New Zealand context. While our data suggest higher cultural efficacy may directly or interactively shield Māori from developing lowered self-esteem typically associated with higher BMI, further research could explore the extent to which Māori may find the Western “thin ideal” personally desirable.

## Supporting information

S1 File Analyses with no covariates. S1 Table in S1 File.
(RTF)
