# Supplementary material for: Cultural efficacy predicts body satisfaction for Māori
Source: PLoS One. 2021 Jun 23;16(6):e0253426. doi: 10.1371/journal.pone.0253426 (PMC8221507; doi:10.1371/journal.pone.0253426)
Supplement: S1 File — S1 Table in S1 File. (RTF) [file pone.0253426.s001.rtf]

We additionally duplicated the main analyses with the covariates removed. Results were not significantly changed. We present these results in brief below and in S1 Table. 
Looking first at the regression predicting body satisfaction, the results showed higher BMI was associated with lower body satisfaction, while cultural efficacy was associated with higher body satisfaction. Critically, the interaction between BMI and cultural efficacy was significant. The simple slopes showed that BMI had a weaker association with body satisfaction for those with high (+1SD) cultural efficacy scores (b = -.074, se = 005, t = -16.774, p < .001, 95% CI[-.082, -.065]) compared to those with low (-1SD) cultural efficacy scores (b = -.101, se = .005, t = -20.253, p < .001, 95% CI[-.111, -.092]).
For the self-esteem regression, the results showed that higher BMI was associated with lower self-esteem, and higher cultural efficacy was associated with higher body satisfaction. Furthermore, the interaction between BMI and cultural efficacy was significant. The simple slopes showed that BMI had a weaker association with self-esteem for those with high cultural efficacy scores (b = -.012, se = .003, t = -3.986, p < .001, 95% CI[-.018, -.006]) compared to those with low cultural efficacy scores (b = -.027, se = .003, t = -8.158, p < .001, 95% CI[-.034, -.021]). 


S1 Table
Multiple regression predicting body satisfaction and self-esteem by BMI and cultural efficacy
	Body Satisfaction	Self-Esteem	
	b	â	se	t	p	95% CI	b	â	se	t	p	95% CI	
BMI	-.088**	-.342	.003	-26.383	<.001	-.094	-.081	-.020**	-.119	.002	-8.792	<.001	-.024	-.015	
Cultural Efficacy	.166**	.126	.017	9.772	<.001	.133	.200	.158**	.185	.011	13.834	<.001	.136	.181	
BMI x Cultural Efficacy	.010**	.053	.002	4.140	<.001	.004	.014	.006**	.047	.002	3.486	<.001	.002	.009	
Note. * p < .05, ** p < .001
 
